# Editorial: Community series in antiviral innate immune sensing, regulation, and viral immune evasion: volume II

**DOI:** 10.3389/fimmu.2023.1341193

**Published:** 2023-12-01

**Authors:** Ling Wang, Junji Xing

**Affiliations:** ^1^ Department of Surgery and Immunobiology and Transplant Science Center, Houston Methodist Research Institute, Houston Methodist, Houston, TX, United States; ^2^ Department of Obstetrics and Gynecology, The Second Hospital of Jilin University, Changchun, China; ^3^ Department of Cardiovascular Sciences, Houston Methodist Research Institute, Houston Methodist, Houston, TX, United States

**Keywords:** innate immunity, antiviral immunity, innate immune sensing, immune regulation, viral immune evasion

Innate immunity is the cellular host’s frontline defense against viral infections. It employs pattern recognition receptors (PRRs) to detect viral nucleotides recognized as ‘pathogen-associated molecular patterns’ (PAMPs) (1, 2). Key RNA-sensing PRRs include Toll-like receptors (TLRs), Retinoic Acid Inducible Gene-I (RIG-I)-like receptors (RLRs), NOD-like receptors (NLRs), C-type lectin receptors (CLRs), Protein Kinase R (PKR), and 2’-5’-Oligoadenylate Synthetases (OAS) and many others (3, 4). Moreover, DNA-sensing PRRs include cyclic GMP-AMP synthase (cGAS), interferon gamma-inducible protein 16 (IFI16), DDX41 and others (5, 6). Following the detection of specific viral PAMPs, PRRs trigger the activation of intracellular signaling cascades, ultimately leading to the induction of type I interferons (IFNs), pro-inflammatory cytokines, and antiviral genes through the activation of interferon regulatory factor 3 (IRF3) and nuclear factor kappa B (NF-κB) (2). These processes not only inhibit viral propagation but also activate the adaptive immune system (2). However, viruses have developed numerous strategies to circumvent the host’s innate immune defense, enabling them to persist and establish ongoing infections. Therefore, understanding the mechanisms of antiviral innate immunity and viral immune evasion strategies remains a focal point of research within the field of innate immunity.

This Research Topic “*Antiviral Innate Immune Sensing, Regulation, and Viral Immune Evasion: Volume II*” highlights 14 recent studies that investigate the mechanisms about antiviral innate immune sensing and regulation in the host, and summarize the innate immune evasion strategies employed by viruses.

The innate immune system plays a vital role in defending against viruses and other pathogens by detecting viral PAMPs and activating various antiviral signaling pathways. These pathways must be precisely regulated to achieve effective antiviral responses while preventing dysregulated immune signaling. The COVID-19 pandemic underscores the importance of comprehending the mechanisms of antiviral innate immune sensing and regulation. Zheng et al. explored the link between COVID-19, rheumatoid arthritis (RA), and the cell death process known as pyroptosis, identifying common biomarkers and potential drug targets. Using comprehensive bioinformatics and network pharmacology analyses, they identified caspase-1 as a key gene involved in the inflammatory responses of both diseases. They also found that the drug minocycline could interact with caspase-1, potentially reducing inflammation in COVID-19 and RA patients. Gu et al. identified A20 as a key regulator in mitigating the inflammation caused by Influenza A virus (IAV) infection. They demonstrated that chronic exposure to low-dose lipopolysaccharide (LPS) reduced inflammation by increasing A20 expression, which then enhanced the activity of peroxisome proliferator-activated receptor alpha (PPAR-α) and PPAR-γ, leading to the suppression of the NF-κB signaling pathway and NLRP3 inflammasome. Huang et al. showed the role of the Ect4 protein in the immune response against viral infections in Drosophila. They found that Ect4, an adaptor protein in the Toll pathway, controlled viral load post-Drosophila C virus (DCV) infection by interacting with Stat92E to regulate the induction of STAT-responsive genes. Mahish et al. investigated the role of Toll-like receptor 4 (TLR4) in Chikungunya virus (CHIKV) infection and the host immune response. They revealed that TLR4 facilitated CHIKV attachment and entry into host macrophages, with TLR4 inhibition significantly reducing viral load, pro-inflammatory responses, and improving survival rates in mouse models. Wu et al. explored the relationship between echovirus infection and autophagy, a key component of the host’s defense mechanisms. They found that echovirus infection triggered autophagy, as evidenced by increased expression of LC3-II and autophagosome formation, and altered signaling pathways involved in autophagosome formation, including decreased phosphorylation of mTOR and ULK1 and increased VPS34 and Beclin-1 levels. Sun et al. investigated how toll-like receptors (TLRs), particularly TLR2 and its heterodimers, affected enterovirus 71 (EV71) replication and innate immune activation. They demonstrated that overexpressing human or mouse TLR monomers and TLR2 heterodimers significantly hindered EV71 replication by stimulating interleukin-8 production via activation of the phosphoinositide 3-kinase/protein kinase B (PI3K/AKT) and mitogen-activated protein kinase (MAPK) pathways. The findings highlight that these membrane-bound TLRs play a critical role in antiviral innate immune sensing and regulation of EV71 infection.

Viruses deploy complex tactics to circumvent the host’s innate defenses and sustain infections. Understanding these evasion techniques will advance our knowledge of viral behavior and inform the development of treatments and vaccines. Weerawardhana et al. showed that the 2B protein of Foot-and-mouth disease virus (FMDV) disrupted the IFN-β production by degrading RIG-I and MDA5, two critical sensors in the type I IFN signaling pathway. They found that FMDV 2B induced ubiquitination and proteasomal degradation of RIG-I via E3 ubiquitin ligase RNF125 and led to MDA5 degradation through caspase-3 and caspase-8, thereby reducing IFN-β production. Wang et al. summarized the antiviral innate immune mechanisms triggered by IFN signal transduction pathways in host cells and the immune evasion mechanisms employed by Rift Valley fever virus (RVFV), particularly through its nonstructural proteins (NSs), providing insight into potential drug targets and strategies to combat Rift Valley fever outbreaks. Zhou et al. reviewed the research progress of the conserved herpesvirus protein kinase UL13 in immune escape and viral replication, providing insights into the pathogenic mechanisms of herpesviruses and potential strategies for their immune escape and replication. Zhao et al. reviewed mechanisms employed by senecavirus A (SVA) to circumvent host defenses, including evading pattern recognition receptor signaling, IFN-α/β receptor pathways, interferon-stimulated genes, autophagy, and stress granules, thereby enhancing our understanding of SVA’s pathogenesis and informing the development of antiviral strategies and vaccines. Zhang et al. highlighted the need for further research into the interplay between herpes simplex encephalitis and innate immunity. Wen et al. reviewed how pestiviruses, significant pathogens in livestock, evaded IFN-mediated immune responses, particularly highlighting the roles of their unique glycoproteins Erns, which inhibits IFN production by cleaving viral RNAs, and Npro, which targets the transcription factor IRF-3 for degradation. Hao et al. identified the African swine fever virus (ASFV) protein QP383R as an inhibitor of the cyclic GMP-AMP synthase (cGAS) pathway, a crucial part of the antiviral innate immune response. They found that QP383R suppressed type I IFN production by interfering with cGAS functions including DNA binding, dimerization, and enzymatic activity, thereby enabling ASFV to evade the cGAS-mediated antiviral innate immune response. Liu et al., investigated patients with hepatitis B (HBsAg-negative but HBV DNA-positive) and found that their cellular immune function, indicated by T-lymphocyte subsets and serum cytokines, was superior to that of HBsAg-positive patients, suggesting reduced liver inflammation. Sequencing of the HBV S region in these patients revealed high-frequency amino acid substitutions and immune escape mutations, potentially leading to undetectable HBsAg levels and changes in its antigenicity and secretion.

Finally, we would like to thank all the authors for entrusting us with their discoveries, and all the referees for their careful and insightful reviews. We are confident that the collection of articles in this Research Topic will captivate researchers focused on antiviral innate immunity and viral immune evasion. Insights into the mechanisms of antiviral immune sensing and evasion are poised to inform the creation of vaccines and antiviral therapies, potentially shaping the response to the COVID-19 pandemic and other infectious diseases in the future.

## Author contributions

JX: Conceptualization, Funding acquisition, Writing – original draft, Writing – review & editing. LW: Writing – original draft.
